# Maximum flow approach to prioritize potential drug targets of *Mycobacterium tuberculosis H37Rv* from protein-protein interaction network

**DOI:** 10.1186/s40169-015-0061-6

**Published:** 2015-06-05

**Authors:** Tilahun Melak, Sunita Gakkhar

**Affiliations:** Department of Computer Science, Dilla University, Gedeo, Ethiopia; Department of Mathematics, IIT Roorkee, India

**Keywords:** Centrality measures, Drug-resistance tuberculosis, Essential genes, Proteome network, Resistance genes

## Abstract

**Background:**

In spite of the implementations of several strategies, tuberculosis (TB) is overwhelmingly a serious global public health problem causing millions of infections and deaths every year. This is mainly due to the emergence of drug-resistance varieties of TB. The current treatment strategies for the drug-resistance TB are of longer duration, more expensive and have side effects. This highlights the importance of identification and prioritization of targets for new drugs. This study has been carried out to prioritize potential drug targets of *Mycobacteriumtuberculosis H37Rv* based on their flow to resistance genes.

**Methods:**

The weighted proteome interaction network of the pathogen was constructed using a dataset from STRING database. Only a subset of the dataset with interactions that have a combined score value ≥770 was considered. Maximum flow approach has been used to prioritize potential drug targets. The potential drug targets were obtained through comparative genome and network centrality analysis. The curated set of resistance genes was retrieved from literatures. Detail literature review and additional assessment of the method were also carried out for validation.

**Results:**

A list of 537 proteins which are essential to the pathogen and non-homologous with human was obtained from the comparative genome analysis. Through network centrality measures, 131 of them were found within the close neighborhood of the centre of gravity of the proteome network. These proteins were further prioritized based on their maximum flow value to resistance genes and they are proposed as reliable drug targets of the pathogen. Proteins which interact with the host were also identified in order to understand the infection mechanism.

**Conclusion:**

Potential drug targets of *Mycobacteriumtuberculosis H37Rv* were successfully prioritized based on their flow to resistance genes of existing drugs which is believed to increase the druggability of the targets since inhibition of a protein that has a maximum flow to resistance genes is more likely to disrupt the communication to these genes. Purposely selected literature review of the top 14 proteins showed that many of them in this list were proposed as drug targets of the pathogen.

**Electronic supplementary material:**

The online version of this article (doi:10.1186/s40169-015-0061-6) contains supplementary material, which is available to authorized users.

## Background

*Mycobacterium tuberculosis* (*Mtb*) which is the etiological agent of tuberculosis (TB) remains one of the main health threats of human by being the cause of morbidity and mortality to millions every year [1]. According to the estimates of WHO global tuberculosis report of 2014, there were 9 million people who developed TB and 1.5 million who died from the disease in 2013 [2]. One of the major factor which makes the management, control and eradication programs of the diseases challenging is the emergence of multi-drug resistance tuberculosis (MDR-TB) and extensive drug resistance tuberculosis (XDR-TB) [3, 4]. MDR-TB is resistance to at least isoniazid and rifampicin which are the two most powerful anti-TB drugs [5, 6]. XDR-TB, in addition to being resistance to at least isoniazid and rifampicin, is also resistance to at least three of the six main classes of second-line drugs (SLDs) [7]. The WHO estimate showed that 3.6 % of the new and 20.2 % of previously treated TB cases have had MDR-TB and an estimated of 9.0 % of patients with MDR-TB had XDR-TB in 2013 [2]. Various strategies have been widely implemented to tackle the problem of resistance in the past including: rotation of antibiotic combinations, identification of less mutable targets, search for new chemical entities for known targets, use of virulence factors as targets and 'phenotypic conversion', which aims to inhibit the resistance mechanism employed by the bacterium [8]. Even with the existence of these important counter measures, the available statistics indicates that resistance forms are still on the rise [9]. Moreover, SLDs are being used for the current treatments of MDR-TB [10]. These treatments are of longer duration, less effective, more toxic, and more expensive than isoniazid and rifampicin. This shows the importance of further investigations to identify reliable therapeutic targets and prioritize the existing ones for the discovery of new drugs.

Various computational methods were used to identify drug targets for the most studied strain of TB, *Mycobacterium tuberculosis H37Rv*. Structure-based drug target identification is one of these approaches [11, 12]. Its drawback is limited availability of protein 3D structures. Another common computational method uses global centrality measures such as closeness/betweenness on molecular interaction networks to identify the targets. A node with higher value of closeness/betweenness centrality would be considered as initial candidates for drug targets [13]. For instance, list of proteins were identified as potential drug targets of *Mycobacterium tuberculosis H37Rv* through network centrality analysis on protein-protein interaction network of the pathogen [14]. These proteins were also non-homologous to human. The global network centrality measures are mainly based on shortest paths without considering other paths that could be important to spread information in the cellular network [11]. This is the common critic with respect to these measures but there are counter arguments. One of which is shortest paths yield a higher coverage than observed directly neighbours locally from protein-protein interaction data. Moreover, it has been hypothesised that shortest paths are the most feasible paths that can be taken by proteins to communicate with each other [9]. Measure of betweenness centrality based on random walks has been suggested as improved versions of these measures [15]. Even though it still gives more weight to the short paths, it tries to incorporate all paths between nodes. There are also computational studies and databases which use integrated approaches with series of filters to identify new drug targets for the pathogen [16, 17]. All of these methods didn’t consider the influence of newly identified targets to drug resistance genes of first line and SLDs to tackle the problem of resistance. However, there are efforts to understand emergence of resistance mechanisms from wholistic system perspective in which co-targets have been suggested for effective antibacterial drugs [9, 18, 19]. The main idea is that the target-co-target combination disrupts the network to reduce the emergence of drug resistance thus allowing the main drug to kill the bacteria. In spite of the importance of co-targets to tackle the emergence of drug resistance, it is not helpful in treating already developed drug resistance. That is one of the main reasons why we need new primary targets than co-targets to tackle this problem.

In this analysis, maximum flow approach has been proposed to further prioritize potential drug targets of *Mycobacterium tuberculosis H37Rv* based on their flow to resistance genes of existing drugs. Since this approach is based on the flow, it is not expected to be affected by biasness towards shortest paths like the common global centrality measures. More importantly, the inhibition of a protein which has a maximum flow to the resistance genes of the existing drugs is expected to disrupt the communication to these genes. So, this further prioritization is believed to be a rational approach to deal with the problem of resistance at the initial stage of drug discovery process.

## Methods

A set of highly reliable potential drug targets P and disease resistance genes set G of *Mycobacterium tuberculosis H37Rv* have been taken as input. The modified version of Melak and Gakkhar [14] has been used to identify potential drug target set P. They were obtained from a comprehensive comparative genome and network centrality analysis. The complete genome of *Mycobacterium tuberculosis H37Rv* was retrieved from Tuberculosis database [20, 21]. BLAST search of the retrieved protein coding genes was carried out against Database of Essential Genes (DEG) to identify proteins which are essential for the survival and growth of the pathogen [22–24]. The resulted proteins were then compared against the non-redundant database with an e-value threshold cut off set to 0.005 [25]. The search was also restricted to H. sapiens because the objective was to find only those proteins, which do not have detectable human homologues to prevent host toxicity. The refined proteins obtained from comparative genome analysis were prioritized and a set P of 137 proteins have been identified. The prioritization was based on network centrality measures and these proteins found at the centre of gravity of the proteome network. A protein is said to be at the centre of gravity of a functional network of interest if its betweenness measure is above the total number of shortest paths expected to pass through it [26]. This cut off has been effectively used by the authors in identification of drug targets.

The drug resistance genes list G is a curated list of 82 genes involved in both intrinsic and extrinsic drug resistance mechanisms of *Mycobacterium tuberculosis H37Rv* identified from two published literatures [17, 18]. In those analyses, they scanned the available biological literatures to obtain information about associations of individual proteins with drug resistance and verified manually to include in the drug resistance list.

Subsequently weighted protein-protein interaction network of the pathogen was constructed by using a dataset retrieved from Search Tool for the Retrieval of Interacting Genes/Proteins (STRING) database. STRING is a dedicated resource of physical and functional protein-protein interactions [27]. It acts as a meta-database which maps all interaction evidence onto a common set of genomes and proteins by integrating information from numerous sources, including experimental repositories, computational prediction methods and public text collections. A combined score is assigned for every pair of protein-protein association in the database [28]. This score is computed by combining the probabilities from several pieces of evidence and correcting for the probability of randomly observing an interaction. The higher combined score value for interacting pair of proteins means the interaction is being supported by several pieces of evidence. It has been shown in a recent comprehensive study that the protein-protein interactions of Mycobacterium tuberculosis H37Rv generated from STRING are of low quality consisting of a significant amount of false positives and false negatives [29]. However, the study also indicated that a subset of this dataset (with combined score ≥ 770) is more reliable with greater portion of it having correlated gene expression profiles and coherent informative Gene Ontology (GO) term annotations in both interaction partners. Thus, this subset of interaction dataset has been used in this analysis and the combined score of the pair of interacting proteins has been assigned as a weight of interaction [30]. It is hypothesized that a higher weight value for a pair of interacting proteins denotes there would be more flow. On the other hand, the weight zero means that the two proteins are not interacting pairs so no flow can pass through them.

In order to understand the general functional organization of interacting proteins, the statistical properties of the generated proteome network were characterized by different measures such as; degree distribution, characteristic path length and clustering coefficient.

One of the most basic properties of a vertex *n*_*i*_ is its degree*k*_*i*_, defined as the number of edges adjacent to the vertex. The degree distribution p (k) is the measure of the proportion of edges in the network having degree *k* [31].

The distance*d*_*ij*_ between any two vertices *n*_*i*_ and *n*_*j*_ in a network is the length of the *shortest path* between them. It is the minimal number of edges that need to be traversed to travel from vertex *n*_*i*_ to*n*_*j*_. The path between two nodes doesn’t necessarily have to be unique since there could be several alternative paths with the same path length. Thus, the characteristic path length is the average shortest path of overall pairs of nodes in the network with *n* vertices [31].

Clustering coefficient is a measure of the probability of two nodes with a common neighbour to be connected [31]. It is an indicator of local cohesiveness and internal structure of the given network. In a given vertex *n*_*i*_ with *k*_*i*_ neighbours of an undirected network, there exist ${E}_{max}=\frac{k_i\ \left({k}_i - 1\right)}{2}$ possible edges between the neighbors. Thus, clustering coefficient *C*_*i*_ of vertex *n*_*i*_ is the ratio of the actual number of edges *E*_*i*_ between the neighbors to the maximal number*E*_*max*_;

$$ {C}_i=\frac{2{E}_i}{k_i\ \left({k}_i - 1\right)} $$

Mean clustering coefficient is then computed as the average of clustering coefficients of all vertices.

The main focus of this analysis is to further prioritize potential drug targets of *Mycobacterium tuberculosis H37Rv* based on their maximum flow to resistance genes. This has been done through identifying the maximum flow between the candidate proteins P and disease genes G. Maximizing the flow from the candidate proteins to the resistance genes seems to be reasonable since understanding the efficient communications in the biological networks can be helpful to design treatment mechanisms for the problem of resistance in a systematic and rational way. The following are the constraints applied to specify the problem into the classical maximum flow problem [11]:

### Constraint 1

The protein-protein interaction is a bi-directional edge.

In the protein-protein interaction network, flow direction is one of the most important features. However, almost all the outcomes of current high-throughput techniques for protein-protein interactions mapping are usually supposed to be non-directional. Thus, the protein-protein interaction in this analysis has been considered as bidirectional edge.

### Constraint 2

A dummy sink node is created to connect all resistance genes with the capacity of these edges set to infinity.

### Constraint 3

All candidate proteins are connected to a dummy source node and the capacity of these edges are set to infinity.

Since infinite value is not practical, the value greater than the maximum possible flow which is the product of maximum degree and maximum capacity has been assigned.

### Constraint 4

If a resistance gene is also a candidate protein, to avoid unfair advantage, it has only been connected to dummy source node but not to the dummy sink node.

Let V be a set of nodes representing proteins, E be a set of edges representing interactions between proteins, and the nodes *s* and *t* be the source and sink nodes respectively. Further, *c* is a nonnegative capacity of the edges. Then, for a weighted interaction network G = (V, E, *s*, *t, c*), the size is denoted as n = |V| and m = |E|. A flow *f* is a real valued function on the edges and the excess value *e*_*f*_ (*v*) is the difference between the incoming and outgoing flows to a node under consideration. A node *v* is said to be active if *v* ∈ *V* − {*s*, *t*}, *d*(*v*) < *n* and *e*_*f*_ (*v*) > 0, and we call an edge (*v*, *w*) admissible if*d*(*v*) = *d*(*w*) + 1. The flow *f* satisfies the following three constraints [32, 33]:

1 $$ \begin{array}{ccc}\hfill \mathrm{Capacity}\ \mathrm{constraint}:\hfill & \hfill \mathrm{f}\left(v,u\right)\le c\left(v,u\right)\hfill & \hfill for\  all\left(v,u\right)\in V\times V\hfill \end{array} $$

2 $$ \begin{array}{ccc}\hfill \mathrm{Skew}\ \mathrm{symmetry}\ \mathrm{constraint}:\hfill & \hfill \mathrm{f}\left(\mathrm{v},\mathrm{u}\right)=\hbox{-} \mathrm{f}\left(\mathrm{u},\mathrm{v}\right)\hfill & \hfill \mathrm{f}\mathrm{o}\mathrm{r}\ \mathrm{all}\ \left(\mathrm{v},\mathrm{u}\right)\in V\times V\hfill \end{array} $$

3 $$ \begin{array}{ccc}\hfill \mathrm{Flow}\ \mathrm{conservation}\ \mathrm{constraint}:\hfill & \hfill {\displaystyle \sum f\left(u,v\right)=0}\ \hfill & \hfill for\  all\ \left(u,v\right)\in V-\left\{s,t\right\}\hfill \end{array} $$

The problem here is to find the maximum flow from the dummy source node to dummy sink node. We wish to achieve that by manipulating the preflow *f* on the network satisfying constraints (1–3).

The following initializations have been made [32]:

The initial value of the flow *f* (*v*, *u*) is set to zero for all (*v*, *u*) ∊ (*V* − {*s*}) × (*V* − {*s*}) and *f* (*s*, *v*) *set to c*(*s*, *v*) for all*v* ∊ *V*.

The distance of a node *d*(*v*)to the sink node *t* is *n* for *v* = *s* and zero for all*v* ∊ (*V* − {*s*}).

The excess flow*e*_*f*_ for the source node is the sum of capacities of all of its edges and zero for all vertices*v* ∊ (*V* − {*s*, *t*}).

Once the initialization is complete, a repeated push and relabel operations have been performed on active nodes starting from the provided source node until there are no more active nodes or the edges are *saturated.*

The *Push* (*v*, *w*) and *Relabel* (*v*) operations:

*Push* (*v*, *w*)*.*

Applicability: v is active and (*v*, *w*) is admissible

Action: send *δ* = *min*(*e*_*f*_(*v*), *u*_*f*_) units of flow from v to w

*Relabel* (*v*)*.*

Applicability: *v* is active and *push* (*v*, *w*) can’t be applied

Action: replace *d*(*v*) by *min*{*d*(*w*) + 1|(*v*, *w*) ∈ *E*_*f*_*or by n if* ∄ (*v*, *w*) ∈ *E*_*f*_}

An edge is said to be *saturated* if the flow on it can’t be increased without violating the capacity constraint and *residual* otherwise [33]. The residual capacity *u*_*f*_ of an edge (*v*, *w*) is the amount by which the flow can be increased. The push and relabel operations modify the preflow *f* and labelling *d*. A push from *v* to *w* increases *f* (*v*, *w*) and*e*_*f*_ (*w*) by $$ \delta =\begin{array}{cc}\hfill min\hfill & \hfill \left({e}_f(v),{u}_f\right)\hfill \end{array} $$, and decreases *f (w, v)* and *e*_*f*_ (*v*) by the same amount. A relabeling of *v* sets its label to the largest value allowed by the valid labeling constraints. FIFO algorithm has been used to maintain the set of active nodes in which the front node is always selected for discharging and the newly active node is added to the rear of the queue [33]. Gap relabeling has been used as a distance relabeling heuristic which is based on the following observation [31]. If we have an integer g and 0 < *g* < *n*. Suppose at a certain stage of the algorithm there are no nodes with distance label *g* but there are nodes *v* with *g* < *d* (v) < *n*. This implies that the sink is not reachable from any of these nodes. Therefore, the labels of such nodes may be increased to *n* (note that these nodes will never be active).

**Fig. 1:**
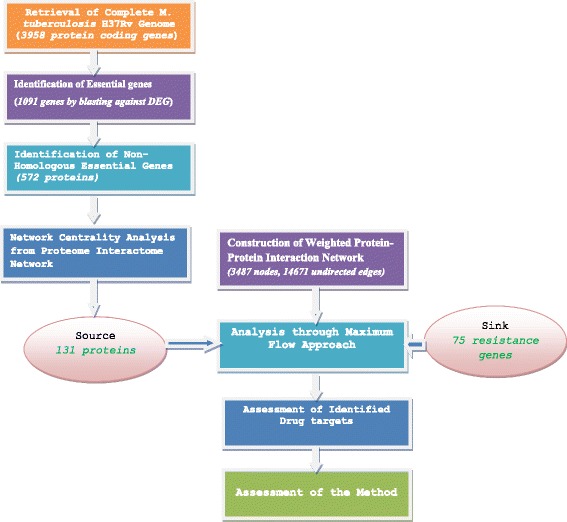
Progression of Experiments

Cytoscape 3.0.2 was used for the generation of statistical properties of the network [34]. It is an open source software platform for visualizing complex networks and integrating these with any type of attribute data. The network centrality measures were computed using CytoNCA, a plug-in of Cytoscape [35]. The maximum flow approach was implemented using adjacency list of First In First Out (FIFO) push-relabel maximum flow with gap relabeling heuristic. For the purpose of proof of correctness, a graph obtained from an analysis by Schroeder et al. (2004) has been used [36]. The progression of experiments of this analysis has been shown in Fig. 1.

## Results and discussion

Discovery of new drugs of TB is one of the solutions to deal with the problem of drug-resistance more systematically [2]. In this study, potential drug targets of *Mycobacterium tuberculosis H37Rv* were further prioritized based on their maximum flow to the resistance genes to identify those targets in which their inhibition would disrupt communications to these resistance genes in the proteome interaction network. The initial input set of potential drug targets of the pathogen, consisting of a list of 137 proteins, is obtained from systematically integrated comparative genome and network centrality analysis. These proteins are believed to be more reliable targets since they are essential for the survival and growth of the pathogen, non homologous to human and found within the close neighbourhood of the centre of gravity of the protein-protein interaction network of the pathogen. There are four types of mechanisms of resistance for *Mycobacterium tuberculosis H37Rv*: efflux pumps, target-modification, DNA replication and Horizontal Gene Transfer (HGT) [9]. A curated list of 82 proteins involved in these drug resistance mechanisms has also been taken as an input for the study*.* Then, protein-protein interaction network was constructed based on the retrieved associations from STRING [27]. Only interactions with a combined score value ≥ 770 were considered in this analysis since they are verified as more reliable dataset containing less false negatives and false positives [28]. The resulting refined network contains 14,671 interactions among 3,487 proteins.

**Fig. 2:**
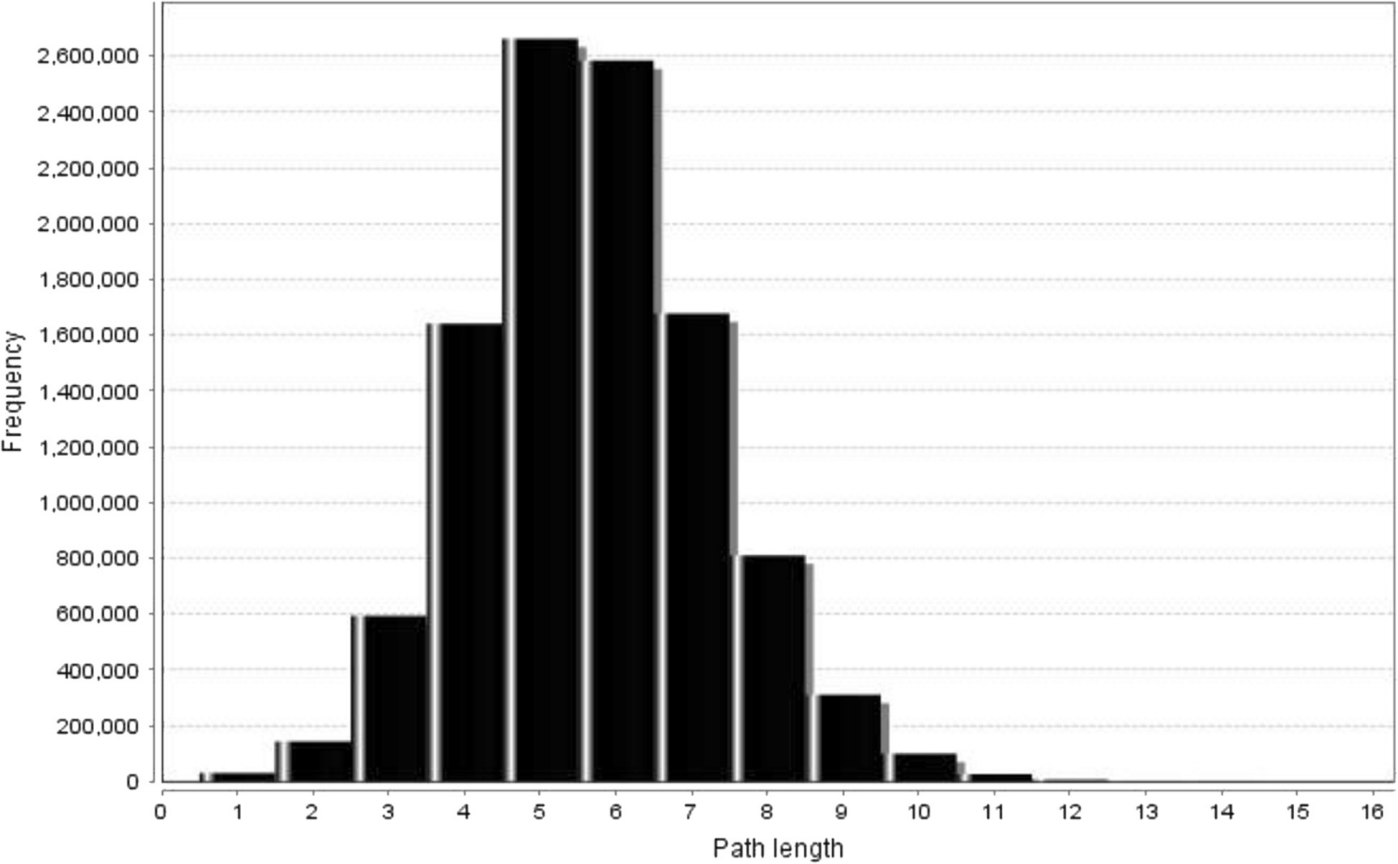
Shortest Path Length Distribution

**Fig. 3:**
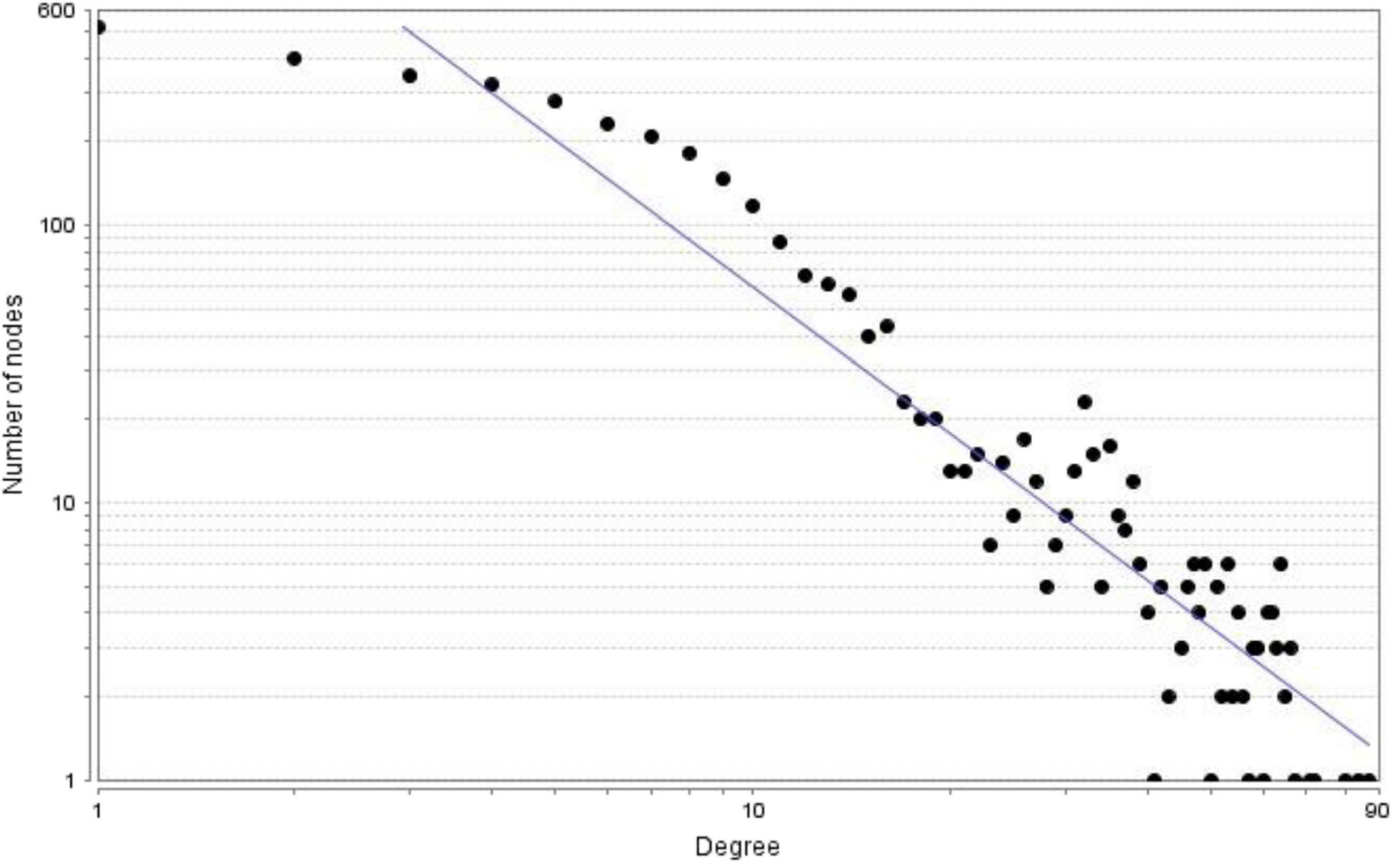
Node Degree Distribution

General statistical properties of the network which is being used to describe essential properties have been shown in Table 1. Characteristic path length is an important property of a network indicating one protein’s influence on another with number of intermediate reactions which would be useful to understand the efficiency of communication of biological information. The corresponding shortest path length distribution has been shown in Fig. 2. Another important property of a network is *clustering coefficient* and the *clustering coefficient* of the resulted network is significantly higher than the *clustering coefficient* of a random graph with the same number of vertices (0.002). The degree distribution of the resulted network has also been shown in Fig. 3. The network exhibits scale-free property like many biological networks in which the degree distribution of proteins approximates a power law *p* (*k*) = *k*^−*γ*^, with the degree exponent *γ* ~ 1.753. This means there are very small number of highly connected nodes called hubs and a vast majority of nodes with few connections.

**Table 1:** Statistical Properties of the Generated Network

Parameter	Value
Number of nodes (n)	3487
Connected components	107
Network diameter	15
Average number of neighbours	8.415
Network density	0.002
Characteristic path length	5.655
Clustering coefficient	0.379

In order to find the maximum flow from the potential drug targets to the resistance genes and prioritize them accordingly, we have defined the source and sink nodes. Out of the 137 proteins taken as potential drug target set, 131 proteins were found in the resulted protein-protein interaction network and these proteins were connected to the dummy source node based on constraint 3. From the curated list of 82 resistance genes, 78 were found in the constructed protein-protein interaction network. Three of them were also candidate drug targets and they have only been connected to dummy source node based on constraint 4. Then the remaining 75 proteins (see Additional file 1) were connected to a dummy sink node based on constraint 2.

The maximum flows of each of 131 potential candidate proteins to the dummy sink node have been identified and they were sorted accordingly. We hypothesize that the flows are used to quantify structural and biochemical signals flow from these candidate proteins to the molecular components of the resistance machinery by which inhibition of the newly proposed targets are expected to have a better success in dealing with MDR and XDR TB. Detail information of the maximum flow of each candidate protein is also provided as an Additional file 2.

In this prioritized list, proteins which interact with the host were identified using a dataset retrieved from a computational prediction of Homo Sapiens- *Mycobacterium tuberculosis H37Rv* protein-protein interactions [37]. This was carried out in order to understand the infection mechanism of the pathogen. As it has been shown in Table 2, 15 out of 131 proteins interact with the host. The reason for the presence of only few overlaps is due to the fact that the host–pathogen interaction dataset is not comprehensive.

**Table 2:** Potential targets interact with human

Protein	Functional class	Max-flow
Rv0732	cell wall and cell processes	49050
Rv1908c	virulence, detoxification, adaptation	33297
Rv1599	intermediary metabolism and respiration	30402
Rv2455c	intermediary metabolism and respiration	27485
Rv2150c	cell wall and cell processes	23472
Rv2534c	information pathways	20846
Rv1712	intermediary metabolism and respiration	20292
Rv2987c	intermediary metabolism and respiration	17639
Rv1415	intermediary metabolism and respiration	16970
Rv3601c	intermediary metabolism and respiration	16574
Rv2553c	cell wall and cell processes	12177
Rv3921c	cell wall and cell processes	12106
Rv1602	intermediary metabolism and respiration	11326
Rv1611	intermediary metabolism and respiration	10147
Rv2538c	intermediary metabolism and respiration	7630

Candidate proteins at the top 10 % maximum flow as lower limit of the 90th percentile which contain 14 proteins (Table 3) were taken for further analysis. The targetablity of a drug target depends on several factors which include: essentiality to the growth and survival of the pathogen, non homologous to the host, availability of 3D structure and gene expression: a target would be desirable if it is expressed in the organism at least under disease conditions. The identified potential targets through this analysis are obviously essential, non homologous to human and found with in close neighbourhood of the centre of gravity of the protein-protein interaction network. Out of the top 14 proteins 5 of them have solved 3D structures. Structures of proteins of the pathogen that don’t have experimentally solved crystal structures can also be obtained using theoretically calculated structural models.

**Table 3:** Top 14 candidate protein drug targets of *Mycobacterium tuberculosis H37Rv*

Gene Symbol	Locus	Functional class	Max-flow	Cross reference (PDB)
rpoA	Rv3457c	information pathways	70861	
rpsC	Rv0707	information pathways	61418	
rplJ	Rv0651	information pathways	61319	
secY	Rv0732	cell wall and cell processes	49050	
gdh	Rv2476c	intermediary metabolism and respiration	36008	
katG	Rv1908c	virulence, detoxification, adaptation	33297	1SFZ;1SJ2;2CCA;2CCD;4C50;4C51;
glcB	Rv1837c	intermediary metabolism and respiration	31666	2GQ3;3S9I;3S9Z;3SAD;3SAZ;3SB0;
hisD	Rv1599	intermediary metabolism and respiration	30402	
korA	Rv2455c	intermediary metabolism and respiration	27638	
rpoZ	Rv1390	information pathways	27485	
gltB	Rv3859c	intermediary metabolism and respiration	26942	
dnaN	Rv0002	information pathways	24226	3P16;3RB9;
sirA	Rv2391	intermediary metabolism and respiration	23588	1ZJ8;1ZJ9;
ftsZ	Rv2150c	cell wall and cell processes	23472	1RLU;1RQ2;1RQ7;2Q1X;2Q1Y;4KWE;

Literature review has been carried out for these top 14 proteins to find out if the result of this analysis is in line with similar studies. All of the top 14 proteins are Tropical Disease Research (TDR) validated targets for *Mycobacterium tuberculosis H37Rv*. TDR Targets database is a dedicated database to facilitate the rapid identification and prioritization of molecular targets for drug development, focusing on pathogens responsible for neglected human diseases [16]. It has also been observed that some of these proteins were reported as potential drug targets of the pathogen by various studies. An integrated approach of an interactome, reactome and genome-scale structural analysis to identify potential drug targets of *Mycobacterium tuberculosis* is a comprehensive approach among these studies which implements multi-step filters. The final list of potential drug targets from this study are likely to be successful since the pipeline incorporates a network analysis of the protein-protein interaction, a flux balance analysis of the reactome, experimentally derived phenotype essentiality data, sequence analyses and a structural assessment of targetablity [17]. From our list of top 14 candidate proteins, secY (Rv0732), katG (Rv1908c), gltB (Rv3859c) and sirA (Rv2391) are among the final list of potential targets identified by this study [17]. katG (Rv1908c) is a validated drug target and multifunctional enzyme, exhibiting both a catalase, a broad-spectrum peroxidase, and a peroxynitritase activities. secY (Rv0732) is significantly more useful as a drug target since it has been involved in the emergence of resistance in the interactome by mediating the flow of information from the existing drugs to the resistance machinery. Rv2455c is among the enzymes identified as drug targets of the pathogen by using in silico analysis of Metabolic Pathways [38]. DNA polymerase III β sliding clamp’s ability to function with diverse DNA repair proteins and cell cycle-control proteins make it a potential drug target [39]. ftsZ (Rv2150c), a bacterial tubulin homologue involved in essential cell division, is considered as an attractive target to develop novel anti-TB drugs, as well as new broad-spectrum antibacterial agents [40].

**Fig. 4:**
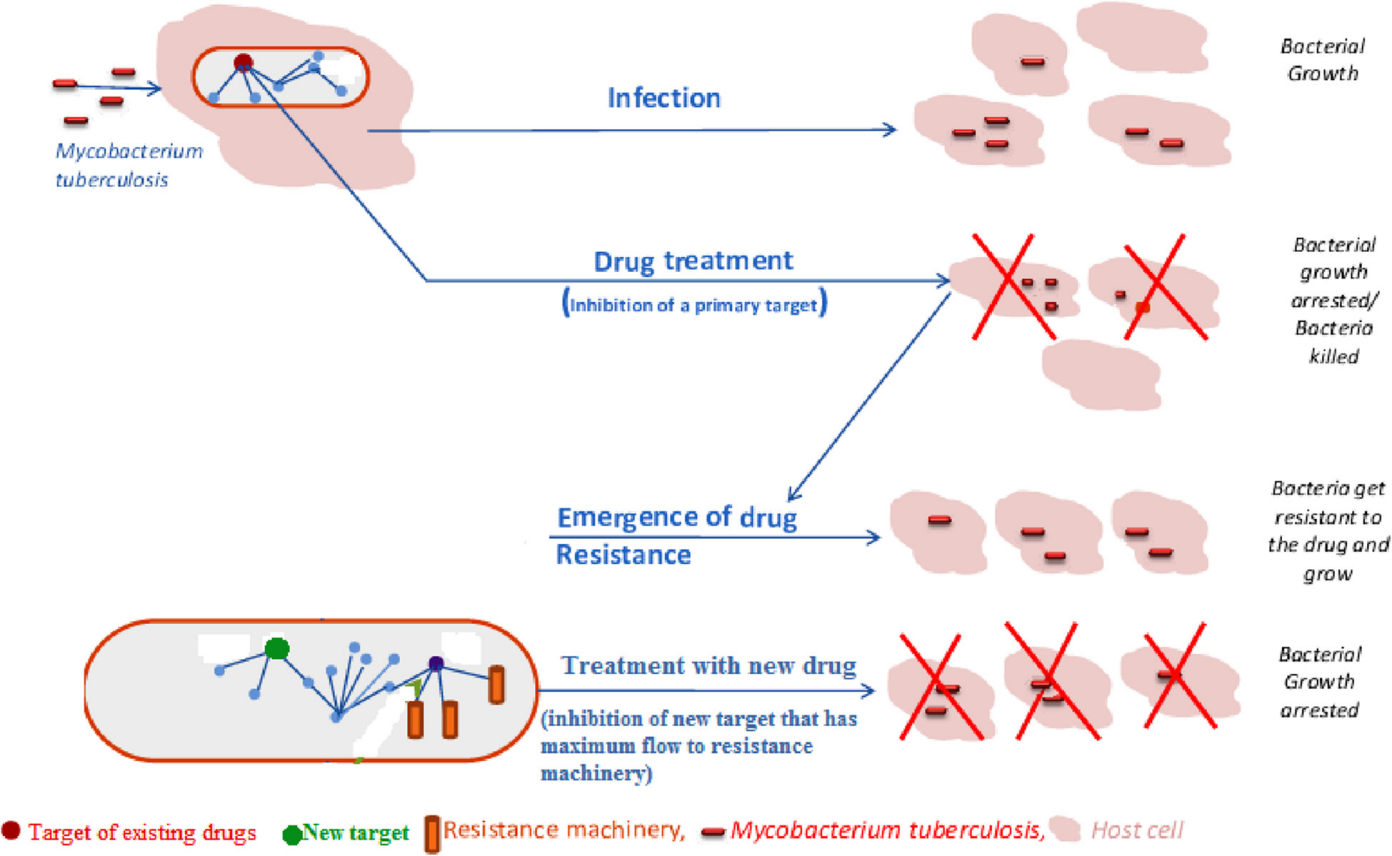
Schematic diagram to depict the proposed mechanism to tackle the problem of resistance (adopted from Raman et al. (2008) [9])

The schematic diagram which depicts the concept about the mechanism of resistance to the existing drugs and the way to tackle the problem with the newly proposed targets has been shown in Fig. 4. Decades-old drugs are no longer effective in killing drug resistance mycobacterium tuberculosis which leads to the requirement of new targets to tackle the problem. The newly proposed targets in this analysis are central to interactome network, essential to the growth and survival of the pathogen, and have higher maximum flow value to resistance genes which increases the druggability since the inhibition of the target would disrupt the communication.

### Assessment of the method

Validation is one of the challenges in identifying drug targets of TB using computational methods mainly due to the unavailability of standard dataset. It would also be ideal to have negative dataset in order to assess their performances. However, such data is not available because of the lack of interest of researchers in validating them [11]. Yeh et al. [11] evaluated the performance of maximum flow approach in identifying drug targets of prostate cancer. This has been carried out through a comparison with other approaches: degree, network entropy, betweenness, closeness and random walk [11]. They obtained the highest mean average precision for maximum flow approach which indicates the method has a better performance than the stated approaches.

In this analysis, validated drug targets for currently existing drugs and essential non- homologous proteins were taken to assess the performance of the method. The 8 clinically used drugs for the treatment of tuberculosis interact with 35 different proteins in the proteome network of the pathogen [18]. Out of these proteins, 34 of them were found in the generated proteome network and they were taken as validated drug targets. From the comparative analysis, 572 proteins were identified. Out of them 537 found in the generated network and taken as essential and non-homologous proteins. Then, the average ranks of the validated drug targets on the non-homologous essential proteins were computed based on their maximum flow value to resistance genes. A lower average rank indicates a better performance. The absolute count of validated drug targets in the top 1 % of non-homologous essential proteins (practically in the top 5 proteins), in the top 5 % (practically in the top 26 proteins), in the top 10 % (practically in the top 53 proteins), in the top 15 % (practically in the top 80 proteins) and in the top 20 % (practically in the top 107 proteins) were reported (Table 4). For instance, in the top 1 %, the method identified 2 validated drug targets.

**Table 4:** Number of drug targets in top 1, 5, 10, 15 and 20 % of the essential non-homologous proteins

Non-homologous Essential proteins	Number of validated drug targets
1 %(5)	2
5 %(26)	4
10 %(53)	13
15 %(80)	21
20 %(107)	21

## Conclusion

In this study, highly reliable potential drug targets and resistance genes to the existing drugs of *Mycobacterium tuberculosis H37Rv* were taken as an input and maximum flow approach has been used to prioritize these proteins based on the flow value of each protein to resistance genes. The potential drug target proteins taken as an input are essential to the survival and growth of the pathogen, non-homologous to human proteins and found near to the center of gravity of interactome network. Resistance genes are curated list of reported genes which are involved in both intrinsic and extrinsic drug resistance mechanisms of *Mycobacterium tuberculosis H37Rv* [9]*.* Using maximum flow approach as a new method on proteome interactome network of the pathogen has an importance of considering the flow instead of shortest paths like other global network centrality measures. There are many well established criteria for assessing the targetablity of potential drug target proteins of various diseases which include essentiality, non homologous to the host, availability of solved structure and gene expression under disease conditions. However to our knowledge there is no comprehensive effort to incorporate the influence of drug targets on resistance genes of diseases like *Mycobacterium tuberculosis H37Rv* as criteria of druggablity*.* This leads to the possibility of including the influences of newly proposed targets on resistance genes as a new concept to assess the druggability of a target. We successfully prioritised potential drug targets based on their flow to resistance genes of the existing drugs of the pathogen. This increases the success rate of the potential drug targets in the rational drug discovery process. A detail literature review of the top 14 drug targets has also showed that many of these proteins have been suggested as highly reliable targets.

## Additional files

Additional file 1:
**Resistance genes. A curated list of resistance genes involved in both intrinsic and extrinsic drug resistance mechanisms of** ***Mycobacterium tuberculosis H37Rv*** **retrieved from literatures.** The list contains those genes found in generated proteome network but not on potential drug target set. Additional file 2:
**Prioritised drug targets.** A list of candidate drug target proteins of *Mycobacterium tuberculosis H37Rv* sorted by maximum flow values.

## Abbreviations

DEG: Database of essential genes
FIFO: First in first out
GO: Gene ontology
HGT: Horizontal gene transfer
MDR-TB: Multidrug-resistant tuberculosis
*Mtb*: *Mycobacterium tuberculosis*
PDB: Protein data bank
SLD: Second-line drug
STRING: Search tool for the retrieval of interacting genes/proteins
TDR: Tropical disease research
TB: Tuberculosis
XDR-TB: Extensively drug-resistant tuberculosis
